# Prostate cancer risk prediction based on clinical factors and prostate-specific antigen

**DOI:** 10.1186/s12894-023-01259-w

**Published:** 2023-06-03

**Authors:** Taewon Hwang, Hyungseok Oh, Jung Ah Lee, Eo Jin Kim

**Affiliations:** 1 Workplace Health Institute, Total Health Care Center, Kangbuk Samsung Hospital, Sungkyunkwan University School of Medicine, B1, 55 Sejong-daero, Jung-gu, 06521 Seoul, South Korea; 2 Department of Economics, Texas A&M University, 4228 TAMU, 77843 College Station, TX USA; 3 Division of Hematology/Oncology, Department of Internal Medicine, Kangbuk Samsung Hospital, Sungkyunkwan University School of Medicine, 29 Saemunan-ro, Jongno-gu, 03181 Seoul, South Korea

**Keywords:** Prostate cancer, Prostate-specific antigen, Prediction model, Lifestyle risk factor, Clinical factor

## Abstract

**Introduction:**

The incidence rate of prostate cancer (PCa) has continued to rise in Korea. This study aimed to construct and evaluate a 5-year PCa risk prediction model using a cohort with PSA < 10 ng/mL by incorporating PSA levels and individual factors.

**Methods:**

The PCa risk prediction model including PSA levels and individual risk factors was constructed using a cohort of 69,319 participants from the Kangbuk Samsung Health Study. 201 registered PCa incidences were observed. A Cox proportional hazards regression model was used to generate the 5-year risk of PCa. The performance of the model was assessed using standards of discrimination and calibration.

**Results:**

The risk prediction model included age, smoking status, alcohol consumption, family history of PCa, past medical history of dyslipidemia, cholesterol levels, and PSA level. Especially, an elevated PSA level was a significant risk factor of PCa (hazard ratio [HR]: 1.77, 95% confidence interval [CI]: [1.67–1.88]). This model performed well with sufficient discrimination ability and satisfactory calibration (C-statistic: 0.911, 0.874; Nam-D’Agostino test statistic:19.76, 4.21 in the development and validation cohort, respectively).

**Conclusions:**

Our risk prediction model was effective in predicting PCa in a population according to PSA levels. When PSA levels are inconclusive, an assessment of both PSA and specific individual risk factors (e.g., age, total cholesterol, and family history of PCa) could provide further information in predicting PCa.

## Highlights

There is a lack of prostate cancer prediction models available in primary care.

This study suggested a 5 year risk model by combining PSA levels and clinical factors.

This model can be used for classifying high risk individuals in clinical practice.

## Introduction

Prostate cancer (PCa) is the second-most common cancer in men worldwide [1]. In Korea, the crude incidence rate of PCa was 58 per 100,000 people in 2018, accounting for 11.5% of all newly diagnosed cancer cases in men [2]. While this is generally lower than numbers from Western countries, the incidence rate has continued to rise in Korea [2]. This is often attributed to the increase in life expectancy and health risk behaviors such as smoking, Western dietary patterns, and sedentary lifestyles, but also to the increased monitoring of serum prostate-specific antigen (PSA) levels [3].

Given the strong association between PSA levels and PCa, PSA is considered an important biomarker for PCa screening [4]. However, PSA is not a cancer-specific marker and can be elevated in non-malignant prostatic conditions such as benign prostate hypertrophy and prostatitis [5]. Other limitations of the PSA test are related with its diagnostic cut-off points. The number of false-positive or false-negative results could increase unnecessary biopsies. To improve the efficacy of assessing PSA in predicting PCa risk, previous studies have proposed risk assessment tools based on additional measurable factors such as free PSA, precursor of PSA, and the prostate health index [6]. These factors can improve the specificity of PCa screening and lead to a more accurate estimation of PCa risk [6, 7]. However, there are few PCa risk prediction models available for utilization in a primary healthcare or community health setting [8].

Distinguished from risk models based on biomarkers, few studies have incorporated epidemiological lifestyle risk factors for PCa risk modeling in the Korean population [9, 10]. Even without the inclusion of PSA levels, these models reported satisfactory performance. However, studies that combine multiple lifestyle factors and PSA levels in risk prediction are scarce, even though PSA levels are widely used biomarkers for PCa screening. Therefore, we aimed to investigate the performance of PCa risk models with the inclusion of PSA levels as a risk factor. In particular, we attempted to develop a clinically useful PCa prediction model when PSA levels are inconclusive, especially in a primary care setting.

## Materials and methods

### Data and study population

In South Korea, the Industrial Safety and Health Law mandates regular health screening examinations for all employees at no expense. Our study utilized data from the Kangbuk Samsung Health Study cohort, which is comprised of Korean men and women aged ≥ 18 years who received an annual/biennial health examination at one of the Kangbuk Samsung Hospital Total Healthcare Centers in Seoul or Suwon, South Korea [11]. We matched the participant data with the Korean Central Cancer Registry provided by the National Cancer Center of Korea to identify patients with PCa up to 2019. This study identified PCa incidences as “C61 (Malignant neoplasm of prostate)” based on the 10th edition of the International Statistical Classification of Diseases and Related Health Problems (ICD-10). This study complied with the Declaration of Helsinki and was approved by the Institutional Review Board of Kangbuk Samsung Hospital, which waived the requirement for informed consent because of the use of anonymized data routinely collected as part of a health checkup program linked to mortality data from the Korea National Statistical Office (IRB No. 2011-01-030-005 for the general Kangbuk Samsung Health Study protocol and 2021-08-046 for the present study).

**Fig. 1:**
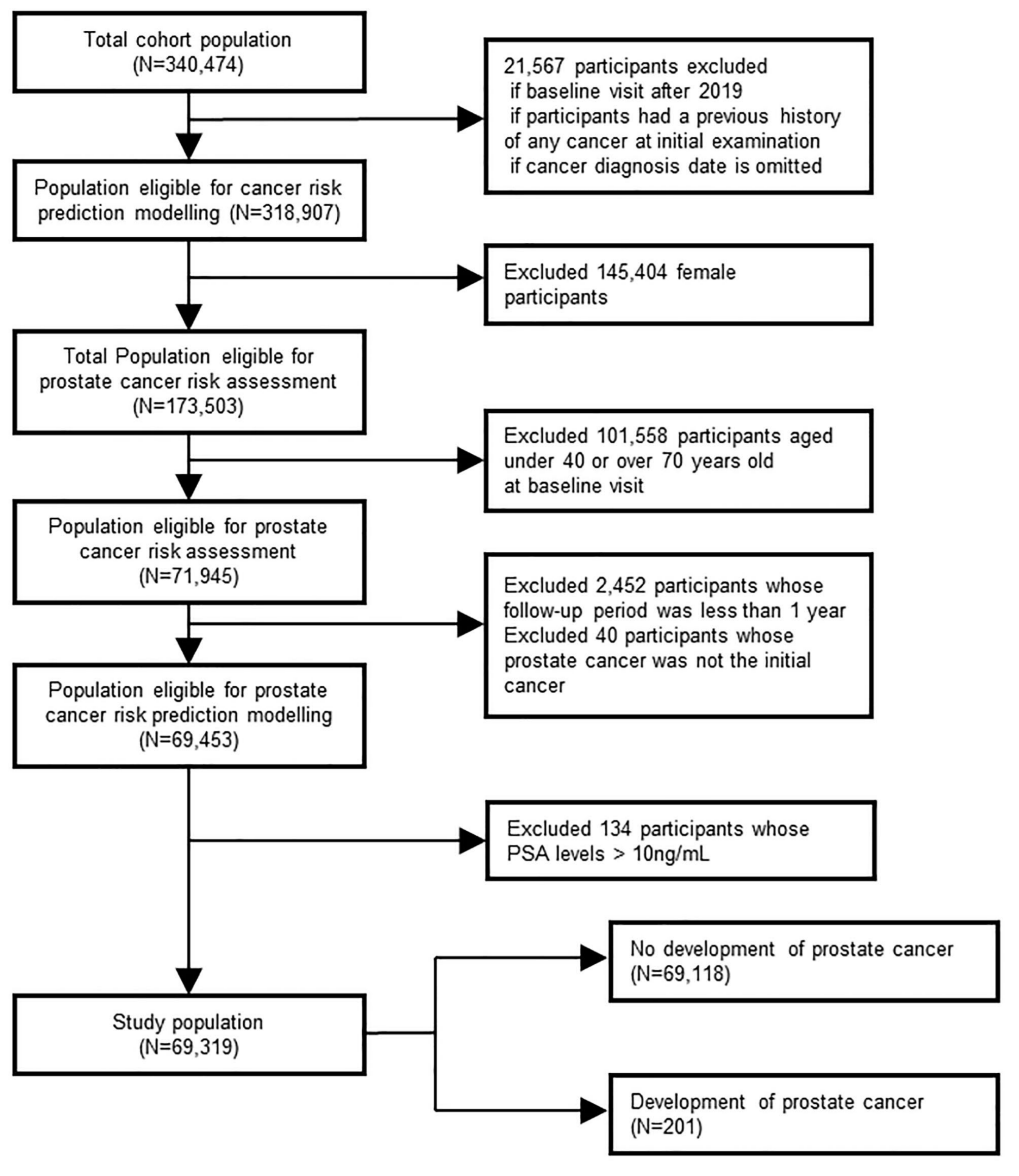
Flowchart of the study population

To construct risk prediction models for PCa, we included men between the age of 40 to 70 who had their initial health examination between January 1, 2011 and December 31, 2018 based on the US Preventive Services Task Force (USPSTF) recommendation against screening in men aged ≥ 70 years [12]. To assess the risk factors associated with the initial diagnosis of PCa, we excluded participants with a history of cancer at their baseline visit or who were initially diagnosed with any other type of cancer. Further, we excluded participants whose follow-up period was less than 1 year after cohort enrollment to reducing the possibility of preexisting prostate cancer at the time of cohort enrollment. Finally, we excluded individuals with elevated PSA levels (> 10 ng/mL) at baseline because they were likely to be strongly recommended by their clinician for further screening. Ultimately, 69,319 participants were eligible for this study, of whom 201 had registered incidences of PCa (Fig. 1).

Data on behavioral factors and medical history were collected using a standardized, self-administered questionnaire included in the health examination. A family history of cancer, past medical history of dyslipidemia, alcohol consumption, and smoking status were collected from the questionnaire. Current smokers were defined as individuals who had a lifetime history of smoking 100 or more cigarettes and were currently smoking. Former smokers were defined as individuals who had a lifetime history of smoking 100 or more cigarettes but were “not at all” currently smoking. Alcohol consumption behavior was categorized based on the frequency and amount of alcohol consumed per drinking day; we classified alcohol consumption in two categories: non- or light drinking, and heavy drinking (< 20, ≥ 20 g/day).

Participants with hyperlipidemia risk were defined as those with high total cholesterol (≥ 200 mg/dL) or those currently on medication for dyslipidemia. For this study, we included PSA levels with the intent of reflecting the incremental risk of PCa according to increasing PSA levels [9, 13]. The Laboratory Medicine Department at the Kangbuk Samsung Hospital in Seoul, Korea has been accredited by the Korean Society of Laboratory Medicine, the Korean Association of Quality Assurance for Clinical Laboratories and the Collage of American Pathologists Survey Proficiency Testing [14].

### Statistical analysis

A conventional Cox proportional hazards regression model was used in this study to develop the PCa risk prediction model. Model performance was evaluated based on the criteria of discrimination and model calibration. To allow for sufficient evaluation of our model, the full dataset was split at a ratio of 8:2, the former to be used for model development (the development cohort) and the latter to be used for model validation (the validation cohort). 5-fold cross validation was performed to evaluate model performance. C-statistics of both the development and validation cohorts were derived to evaluate discrimination. The Nam-D’Agostino test was used to evaluate the calibration of the model in each cohort [15]. We selected variables based on previous well-known risk factors including age, smoking status, alcohol consumption, hypercholesterolemia, and family history of PCa. The potential risk factors included in our final multivariable models were age, smoking status, alcohol consumption, past medical history of dyslipidemia, family history of PCa, and PSA levels.

The estimating equation for the probability of developing PCa within *t* years is given as follows:

$$ P\left(PCa\right)=1-{h}_{0}{\left(t|t=T\right)}^{\text{e}\text{x}\text{p}\left(\sum _{i=1}^{I}\widehat{{\beta }_{i}}*{x}_{i}\right)}$$

where ${x}_{i} (i=1,\dots , I)$ refers to risk factor *i*, $\widehat{{\beta }_{i}} (i=1,\dots ,I)$ refers to the estimated coefficients from the Cox proportional hazards models, and ${h}_{o}\left(t\right|t=T)$ refers to the baseline survival estimate at time *t*. Given the time spread of the data, we estimated the risk of developing PCa in five years (*T* = 5).

The model was validated based on general indices of discrimination and calibration. Harell’s C-statistics were calculated to assess the models’ accuracy in ranking those with higher likelihood of developing PCa. The receiver operating characteristic (ROC) curves provided intuitive insight into the predictive ability of the two models based on the occurrence distribution at *T* = 5. The calibration was measured using the Nam-D’Agostino test, executed as follows: ten subgroups were generated based on the estimated risk of PCa, and the proportion of observed occurrence and the average estimated predictions were compared using a ${\chi }^{2}$ distribution. Hence, the Nam-D’Agostino test statistic allows the assessment of whether predicted values adhere to the observed occurrence data. All analyses were completed using STATA version 17.0 (StataCorp, College Station, TX, USA).

## Results

### Baseline characteristics of study participants

A predominant proportion of the study cohort received their first medical examination at the Kangbuk Samsung Hospital Total Healthcare Centers in their forties. The baseline visits of 15,670 and 5,918 participants were in their fifties and sixties. The incidence of PCa between the age groups showed a sharp increase with age. That is, the incidence per 100,000 person-years was approximately 16 times higher in the 60–70 age group than in the 40–50 age group, totaling an average incidence of approximately 45 per 100,000 person-years. Furthermore, approximately 48% (96 incidences) of PCa diagnoses were made within five years of the initial health examination.

### Evaluation of risk factors for prostate cancer

We showed the descriptive statistics of variables and incidence of PCa at the participants’ first visit in Table 1. We showed the incidence of PCa according to age, the presence of hyperlipidemia, family history of PCa, and PSA levels among the full study cohort. Utilizing these risk factors, we constructed a multivariable Cox proportional hazards regression model using our development cohort. As shown in Table 2, it is evident that PSA is a significant and substantial risk factor associated with increasing hazard ratios for increasing PSA levels. When PSA level increased 1 ng/dL, the HR ratio for PCa increased by 1.774 (95% CI: [1.673–1.881]). Age, inclusion in a hyperlipidemia risk patient group, and family history of PCa, and PSA were statistically significant (*p* < 0.05) risk factors of PCa. Although smoking status and alcohol consumption were not strong risk factors, we included those factors in multivariable analyses since they were assessed to contribute to a better global model fit.

**Table 1:** Baseline characteristics of study participants and incidences of prostate cancer (full cohort)

Risk factor		Frequency			
		Participants at baseline (%)		Incidences (%)	
**Age (yrs)**					
40–50		47,731	(68.86)	51	(25.37)
50–60		15,670	(22.61)	73	(36.32)
60–70		5,918	(8.54)	77	(38.31)
**Smoking status**					
Non-smoker		9,964	(15.42)	32	(17.58)
Former smoker		29,790	(46.09)	99	(54.4)
Current smoker		24,875	(38.49)	51	(28.02)
**Alcohol consumption (g/day)**					
< 20		37,821	(57.15)	104	(58.43)
≥ 20		28,358	(42.85)	74	(41.57)
**The presence of hypercholesterolemia (mg/dl)**					
total cholesterol < 200		31,110	(44.88)	68	(33.83)
≥ 200 or on medication for hyperlipidemia		38,208	(55.12)	133	(66.17)
**Family history of PCa**					
No		64,995	(93.85)	176	(87.56)
Yes		4,259	(6.15)	25	(12.44)
**PSA (ng/mL)**					
< 3.0		67,464	(97.32)	117	(58.21)
3.0-6.9		1,678	(2.42)	69	(34.33)
7.0–10.0		177	(0.26)	15	(7.46)

Abbreviations: PCa, prostate cancer; PSA, prostate specific antigen

**Table 2:** Multivariable regression analysis (development cohort)

Risk factor		HR	[95% CI]	p-value
**(Age-40)**		1.126	[1.102,1.150]	< 0.001
**Smoking status**				
Non-smoker		1	-	-
Former smoker		1.147	[0.691,1.901]	0.596
Current smoker		1.143	[0.652,2.00]	0.642
**Alcohol consumption (g/day)**				
< 20		1	-	-
≥ 20		1.158	[0.821,1.631]	0.403
**The presence of hypercholesterolemia (mg/dl)**				
total cholesterol < 200		1	-	-
≥ 200 or on medication for hyperlipidemia		1.802	[1.257,2.585]	0.001
**Family history of PCa**				
No		1	-	-
Yes		1.648	[0.944, 2.878]	0.079
**PSA (ng/mL)**		1.774	[1.673, 1.881]	< 0.001

Abbreviations: HR, hazard ratio; CI, confidence interval; PCa, prostate cancer; PSA, prostate specific antigen

### Model performance and comparisons

**Fig. 2:**
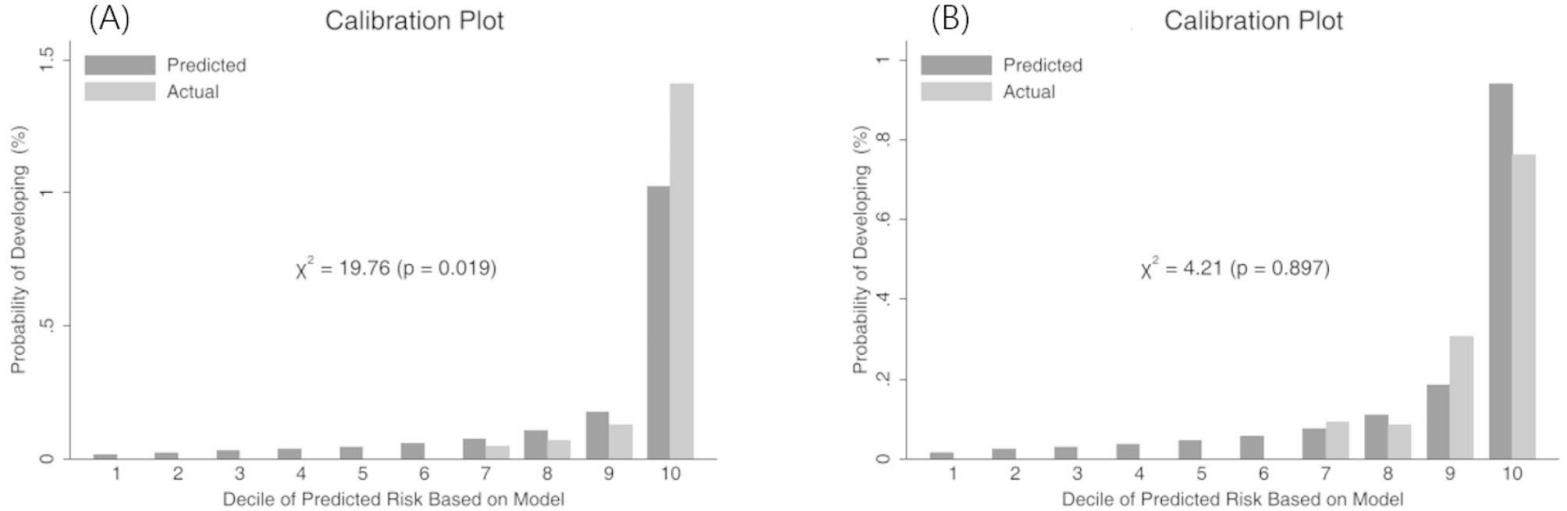
Discrimination and calibration plots of the cancer risk prediction model of the validation cohort. **A**, deciles of predicted and real incidence rates in the validation cohort, **B** deciles of predicted and real incidence rates in the validation cohort. Nam-D’Agostino test statistics are displayed in plots **A** and **B**

**Fig. 3:**
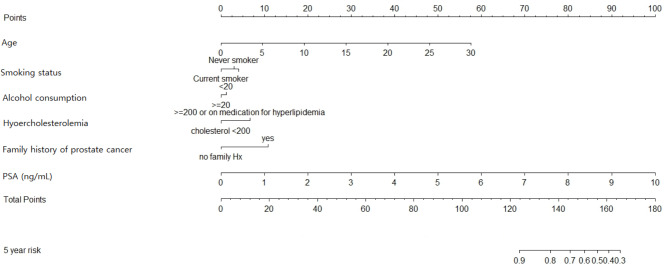
Nomogram for calculating the probability of developing PCa

The general indices of calibration and discrimination were investigated to confirm the validity of the model. The C-statistics for the development and validation cohorts were calculated: 0.922, 0.874 for the development cohort and validation cohort, respectively. From these results, we concluded that the ability of the risk prediction model to accurately rank higher-and-lower risk groups was sufficient. The calibration plot is found in Fig. 2. The Nam-D’Agostino test statistic was 19.76 (*p* = 0.019) and 4.21 (*p* = 0.897) for the development and validation cohorts. These results indicate that our model showed satisfactory calibration with the real PCa occurrence data. Also, we added the nomogram for calculating the probability of developing PCa in Fig. 3.

## Discussion

The present study suggests an effective PCa prediction model that could be practically used in primary care or a community health setting. Our model had sufficient discrimination ability in both development and validation cohorts, suggesting high efficiency in PCa prediction. In addition, our risk prediction model can provide useful information for clinical decisions on whether further evaluation for PCa should be performed when PSA levels are inconclusive.

Most previous prediction models for PCa have focused on the measures of PSA (freePSA, %fPSA, PSAD, p2PSA etc.) that maximized the accuracy of PCa detection [16–22]. These studies achieved high predictive accuracy and area under the curve (AUC) measures. However, the applicability of these models in a clinical setting is limited because they often focused on narrow PSA ranges (2.5-4.0 ng/mL) or on biomarkers less available in primary care or health examinations. On the other hand, few studies on PCa risk prediction investigated individual risk factors associated with PCa carcinogenesis [9, 10, 23]. However, considering that serum PSA is a commonly performed screening test in health examinations, a model consisting of only questionnaire-based risk factors is limited in providing additional information for people with marginal PSA levels.

This study proposed a PCa risk prediction model for clinical implementation by combining PSA levels and PCa risk factors. The efficacy of our model was evaluated in terms of both discrimination and calibration, and we established that our model was sufficient in producing statistically meaningful inferences. From the multivariable model, when PSA level increased 1ng/mL, the risk for PCa development increased 1.7 times higher after adjusting for other risk factors. Combined with other PCa risk factors, our model can provide useful information for clinical decisions on whether further evaluation for PCa should be performed when PSA levels are inconclusive. Moreover, since our model provides estimates based on predictions of 5-year risk of PCa, it may aid in the process of classifying individuals that require further close observation.

We performed the validity of several conventionally contested PCa risk factors including age, smoking status, alcohol consumption, past medical history of dyslipidemia, cholesterol levels, and family history. Aging is a main risk factor for PCa [24]. In our study, we found that a one-year increment in age after 40 years is associated with an approximately 20% increased risk of PCa. We also included age squared with the intent of capturing a quadratic increase in PCa risk with age but found little evidence to substantiate this hypothesis. Smoking status and alcohol consumption are risk factors often investigated for the risk of PCa [25]. Although those factors were not statistically significant in multivariable analysis, they were well-known carcinogen and included in our prediction model.

A family history of PCa appeared to have a significant relationship with PCa carcinogenesis. These findings were consistent with those reported by Lesko et al. (1996) and support the literature on the genetic predispositions of PCa (e.g. BRCA1, BRCA2 mutations) [26]. Finally, our model suggested the presence of hypercholesterolemia as a risk factor, defined by an assessment of total cholesterol (≥ 200 mg/dL) or dyslipidemia medication administration. It was associated with an approximately 67% increase in PCa risk, even after controlling for other factors. Previous studies have reported that elevated cholesterol levels could affect cell proliferation, inflammation, and lipid accumulation in the prostate [27]. The rapid growth of cancer cells might require a sufficient amount of cholesterol [27]. A recent study showed that androgen-independent PCa cell growth could be influenced by extracellular lipid levels and low-density lipoprotein-cholesterol availability [28]. Since many cancers develop as a consequence of chronic inflammation, persistent inflammation induced by cholesterol may increase the risk of PCa.

Our study had several limitations. First, the incidence of PCa was relatively lower than that of the National Cancer Statistics in Korea and it was not associated with several individual risk factors debated in previous studies. Direct comparisons with previous studies are unwarranted, however, because our study participants were younger than the general Korean population. Further, regarding our study cohort, it should be recognized that the participants at the Kangbuk Samsung Total Healthcare Centers tend to be relatively healthier and have higher socioeconomic statuses than the general Korean population [29]. Since the association between socioeconomic status on the prognosis of PCa is notable from previous studies, this aspect may limit the generalizability of this study [16]. Second, the follow-up period may be not sufficient to observe the development of PCa in this study. Hence, the risk of several factors could have been underestimated. Finally, it should be noted that lifestyle attributes, such as smoking status, alcohol consumption, and family history of PCa, were extracted from a self-reported questionnaire. Resulting recall biases may lead to an underestimation of the magnitude of these risk factors. However, as evidence to negate this claim, we found that the association between cotinine levels and smoking status was significant in our data, and further refer to Kerber and Slattery (1997) to suggest that recall bias for PCa is relatively small [30].

Despite the limitations above, we propose that the rich quality and quantity of our data, and the accuracy of the PCa registry data from the National Cancer Center are features of our study that bolster the credibility of our results. Moreover, a prediction model developed in our study are sufficiently effective, suggesting that the potential risk factors that were included in the models are collectively strong predictors of PCa risk. We observed that individuals with increased PSA levels were associated with a significant increase in PCa risk within 5 years. Also, our prediction model can provide useful clinical information to classify high risk population with inconclusive PSA levels.

## Conclusion

Our prediction model can provide useful clinical information to classify high risk population with inconclusive PSA levels. These results are helpful in a clinical setting where PSA screening is less costly and hence frequently practiced. Furthermore, the development of advanced PCa risk prediction models could provide useful information to discriminate high-risk groups of PCa.

## Data Availability

The data are not publicly shared because we do not have permission from the Institutional Review Board to distribute the data. The analytic methods are available from the corresponding authors upon reasonable request.

## Abbreviations

CI: Confidence interval
HR: Hazard ratio
ICD-10: International Classification of Diseases and Related Health Problems 10th Revision
PCa: Prostate cancer
PSA: Prostate-specific antigen
USPSTF: US Preventive Services Task Force
